# Editorial: Green psychology: nature and scope for sustainability

**DOI:** 10.3389/fpsyg.2023.1260680

**Published:** 2023-10-10

**Authors:** Murale Venugopalan, Viswanathan Pozhamkandath Karthiayani, G. Rejikumar

**Affiliations:** ^1^Amrita School of Business, Amrita Vishwa Vidyapeetham University, Kochi, India; ^2^Amrita School of Business, Amrita Vishwa Vidyapeetham University, Kollam, India

**Keywords:** green psychology, human-nature interactions, agriculture, sustainability, stakeholder perceptions

## Background

The interplay between human psychology and the natural environment has gained significant global attention in recent years. Green psychology, such as ecopsychology, is part of environmental psychology and investigates the psychological, emotional, and cognitive effects of nature on individuals and society. It explores the intricate relationship between human beings and nature, emphasizing the psychological benefits and behaviors associated with nature engagement and sustainability outcomes. Extant research has consistently shown that a stronger “human nature connection” is associated with pro-environmental attitudes, conservation and ecological awareness, adoption of sustainable lifestyle choices, and the development of an environmental identity (Klaniecki et al., 2018; Martin et al., 2020; Gansser and Reich, 2023). By recognizing and nurturing this connection, individuals can contribute and foster a sustainable future.

This special issue of articles on the Research Topic (RT) “*Green psychology: nature and scope for sustainability*” includes a good collection of research articles with an overarching objective to explore how green psychology meets the challenge of developing a sustainable society and how it can be applied to design effective interventions that promote sustainable consumption patterns. The RT sought explorations on establishing how to bridge the gap between theory and practice in green psychology-related paradigms to inform policymakers about emerging consumption trends and craft practical solutions to environmental problems. An assessment of how green psychology can contribute to the achievement of the UN Sustainable Development Goals 2030 and help create new models for sustainable behavior for individuals, organizations, and societies was expected to be a central component of such discussions in the articles. Additionally, we expected that by unveiling the emerging thought processes, emotions, beliefs, and attitudes of individuals to adopt green practices, the RT may contribute to the creation of products, services, or practices that mitigate global environmental challenges.

## A synopsis of articles in the Research Topic

The articles included in this RT cover a wide variety of fields outside the specific application of theoretical concepts in many areas related to sustainability outcomes. These articles have contributed to the overall understanding of the subject matter and have advanced our knowledge in the fields of human psychology and its interface with the environment and sustainability. The call for articles has resulted in the receipt of a wide range of submissions, with a final selection of eleven original research articles prompting discussions on defining the concept of green psychology, developing models, theories, and frameworks on its influence on human behavior toward sustainability. This RT is a result of the collaboration between researchers from different parts of the world and focuses on sustainability through farmer behavior in agriculture (8 articles), citizen behavior in climate control (1 article), patient behavior in healthcare (1 article), and consumer behavior in green retail (1 article). The articles are the outcomes of research focused on many countries, such as China, Korea, Malaysia, Germany, and Iran.

As emerges from the articles, understanding the multiple stakeholder perspectives is crucial as they determine green adoption behavior. Stakeholders in a specific sustainable practice need to be motivated and educated to understand the importance of green practices and how they can contribute to sustainability. It can be related to sustainable product design or green prevention and control techniques. Sustainable design includes responsibly using materials that are recyclable or biodegradable, thus minimizing waste. Mostly, ambiguity about stakeholder or end-user emotions and tastes significantly poses challenges to sustainable design practices. The original research included in this RT by Kam and Yoo documents a design practice method to satisfy customer sensibility and individual taste in the fashion industry. The agricultural sector faces many sustainability challenges, and farmers are the most important stakeholder group willing to adopt sustainable farming methods, which are well understood as climate-resilient or climate-smart agricultural practices. In this regard, many articles in this RT address different aspects of farmer perspectives on the adoption of practices that develop sustainable outcomes. This included new technology adoption (Jia et al.), straw returning technology (Ren and Zhong), sustainable certification (Rizal and Nordin), low-carbon agricultural technologies (Jiang et al.), green control technology adoption (Chen et al.; Ren et al.), livestock manure resource utilization behavior (Gao et al.), and rangeland conservation (Savari).

The empirical studies that looked at stakeholder behavior in the adoption of sustainable practices explored several theoretical perspectives and found them relevant in the context of the studies. These include the theory of planned behavior (Ren and Zhong; Jiang et al.), rational-choice theory (Rizal and Nordin), rational behavior theory (Jiang et al.), new economic sociology theory (Ren et al.), information-motivation behavior (Chen et al.), unified theory of acceptance and use of technology (Jia et al.), and the value-belief norm (Savari). A few observations documented in this RT have policy implications. First, they suggest a cognitive process in sustainability adoptions referred to as a “prospective benefit and drawback evaluation,” which involves perceived value, the level of social capital, and the level of subjective cognition (Gao et al.). Second, increasing the social capital of farmers could encourage them to embrace innovative practices (Ren et al.). Effective communication and responsible leadership are beneficial in developing a higher level of participation by smallholder farmers in sustainable practices (Rizal and Nordin). Third, the cognitive deficiencies of farmers are an important barrier to the adoption of sustainable practices, and government assistance is essential to overcome such challenges (Ren and Zhong).

The research by Song et al. examined the psychophysiological restorative potential of cancer patients through virtual reality (VR)-based perception of the natural environment and observed that blue and green hospital environments are more beneficial to psychological health compared to gray. The study established that human wellbeing is positively affected by natural environments, even among cancer patients. Similarly, Blöbaum et al. observed substantial similarities between nature conservation beliefs grounded in a biospheric value orientation (protecting biodiversity) and climate protection values and norms; there did not seem to be value-based conflicts between nature conservation and climate protection.

## Looking forward: advancing green psychology research

As regards the emerging research direction, more recent policy articles and scientific studies indicate a trend toward new topics and technologies, such as carbon capture and storage (CCS), carbon capture and use (CCU), bioenergy with carbon capture and storage (BECCS), direct air carbon capture and storage (DACCS), use of hydrogen, and negative emission technologies (NETs) (Antonini et al., 2020; Borchers et al., 2022). It is important to understand the possible psychological conflicts in terms of drivers and barriers associated with these upcoming technologies, their impacts on nature and landscapes, and the eventual sustainability transformation. Further adoption and scaling up of green psychology interventions, including nature-based therapies, environmental education programs, creation of green spaces in urban environments, and ecotherapy/eco-counseling, could offer valuable approaches for promoting wellbeing, sustainable behaviors, and a deeper connection with nature. These interventions have shown promising results in enhancing mental health, fostering pro-environmental attitudes, and facilitating sustainable behaviors.

From policy perspectives, integrating green psychology into policies and practices is essential across various sectors for promoting sustainability and enhancing individuals' connection with nature. By incorporating principles of green psychology in healthcare, education, urban planning, and organizational management, it may be possible to leverage psychological insights to support pro-environmental behavior change and foster a deeper appreciation for the natural world. Such policy integrations can contribute to a more sustainable future where policies and practices prioritize the wellbeing of both individuals and the environment.

## Author contributions

MV: Writing—review and editing. VK: Writing—review and editing. GR: Writing—review and editing.
